# Numerical Simulation and Experimental Analysis for Microwave Sintering Process of Lithium Hydride (LiH)

**DOI:** 10.3390/ma17215342

**Published:** 2024-10-31

**Authors:** Yuanjia Lu, Maobing Shuai, Jiyun Gao, Xiaolei Ye, Shenghui Guo, Li Yang, Bin Huang, Jiajia Zhang, Ming Hou, Lei Gao, Ziqi Zhou

**Affiliations:** 1 Faculty of Metallurgical and Energy Engineering, Kunming University of Science and Technology, Kunming 650093, China; luyuanjia98@163.com (Y.L.); stoneye2@163.com (X.Y.); 20040051@kust.edu.cn (S.G.); houmingkmust@163.com (M.H.); zhouzq0128@163.com (Z.Z.); 2 State Key Laboratory of Complex Nonferrous Metal Resources Clean Utilization, Kunming University of Science and Technology, Kunming 650093, China; 3 Science and Technology on Surface Physics and Chemistry Laboratory, Jiangyou 621908, China; shuaimaobing@caep.cn (M.S.); huangbin1007@126.com (B.H.); zhangjia200512@126.com (J.Z.); 4 School of Chemistry and Environment, Yunnan Minzu University, Kunming 650093, China; jiyungao89@163.com

**Keywords:** LiH, microwave sintering, dielectric properties, finite element simulation

## Abstract

Dense lithium hydride (LiH) is widely used in neutron shielding applications for thermonuclear reactors and space systems due to its unique properties. However, traditional sintering methods often lead to cracking in LiH products. This study investigates the densification sintering of LiH using microwave technology. A multiphysics model was established based on the measured dielectric properties of LiH at different temperatures, allowing for a detailed analysis of the electromagnetic and thermal field distributions during the microwave heating of cylindrical LiH samples. The results indicate that the electric field distribution within the LiH is relatively uniform, with resistive losses concentrated primarily in the LiH region of the microwave cavity. LiH rapidly absorbs microwave energy, reaching the sintering temperature of 520 °C in just 415 s. Additionally, the temperature difference between the low- and high-temperature regions during the sintering process remains below 5 °C, demonstrating excellent uniform heating characteristics. The microwave sintering process enhances interface migration within the LiH samples, resulting in dense metallurgical bonding between grains. In summary, this research provides valuable insights and theoretical support for the rapid densification of LiH materials, highlighting the potential of microwave technology in improving material properties.

## 1. Introduction

Lithium hydride (LiH) is a high-energy-density ceramic material with broad applications in nuclear industry, organic synthesis, and batteries [1,2,3,4,5,6]. Its high energy density and excellent hydrogen storage properties render it promising for energy storage and conversion [7,8,9]. In designing neutron shielding for thermonuclear fuels and space reactors, LiH components typically need to be large and thick to be effective in practical applications. However, the unique physicochemical and metallurgical properties of LiH pose a challenge for fabricating high-quality, large-sized components that meet these requirements.

Traditional fabrication methods for large LiH components, such as casting and conventional sintering techniques, face numerous limitations in equipment and processes. The traditional sintering method involves placing materials in a high-temperature furnace, where heat is transferred to the interior of the material through conduction, facilitating the bonding of powder particles. Traditional sintering is limited by the heat transfer process, resulting in non-uniform temperature distribution within the material during heating and cooling [4,10,11]. This non-uniformity creates temperature gradients, particularly pronounced in large-sized LiH components. The temperature gradient indicates that different areas of the LiH component are heated unevenly, which generates uneven thermal stresses. Concentrated thermal stress can cause micro-cracks in the LiH material, which may propagate during subsequent sintering, leading to macro-scale cracking of the component [10,12,13]. Such cracking not only affects the structural integrity of the component but also weakens its mechanical performance, thereby impacting its reliability in practical applications [14]. Therefore, it is essential to investigate and develop innovative sintering technologies to address these inherent issues, enhancing the quality and performance of thick-walled LiH products.

Microwave sintering is an innovative sintering technique that employs microwave radiation to directly heat materials. The fundamental principle involves using the microwave electromagnetic field to induce vibrations in the molecules or ions within the material, thereby generating heat. Microwave sintering technology, an advanced method utilizing microwave electromagnetic fields for heating and sintering materials, offers advantages over traditional sintering processes. These advantages include rapid heating, low energy consumption, uniform temperature distribution, and lower sintering temperatures. For large components, microwave sintering allows for efficient rapid heating and uniform densification, reducing sintering temperatures and times while improving material density and mechanical properties [15,16,17]. For instance, Chen et al. reported the microwave sintering of ultra-large ZTA ceramics, achieving a maximum hardness of 14.2 GPa with a sintering time of approximately 4 h, which is markedly superior to traditional methods [18]. Similarly, Manière et al. utilized electromagnetic thermodynamics simulations and experiments to fabricate large-sized, complex-shaped samples, revealing the advantages of microwave sintering in material homogeneity and grain growth control. Microwave sintering has shown great potential in the rapid densification of thick-walled LiH components [19]. However, due to the unique nature of LiH materials, research on their microwave sintering under such conditions is relatively scarce. Additionally, the closed environment of the sintering process and the rapid microwave heating make it challenging to directly observe temperature uniformity during microwave heating. Therefore, finite element simulation is an effective method to analyze the temperature rise behavior of LiH under microwave heating [20]. Through finite element simulation, detailed predictions and analyses of the temperature distribution during the heating process of LiH can be made, aiding in optimizing microwave heating processes and improving the quality and performance of the components.

The finite element method (FEM) is a numerical analysis technique commonly used to address complex physical problems. By discretizing the research object into a finite number of elements, FEM can effectively simulate complex geometric structures and multi-physics interactions. In microwave heating studies, FEM can model the interaction between electromagnetic fields and thermal fields, thus predicting the material’s temperature distribution [21,22]. Applying FEM to the microwave sintering of LiH enables a detailed analysis of its temperature response under microwave electromagnetic fields, revealing temperature distribution patterns and heat transfer mechanisms during heating. For instance, Bhoi et al. developed an innovative integrated approach for aluminum microwave hybrid sintering using FEM, demonstrating good agreement between simulated and experimental temperature variations (within 10%) [23]. Putra et al. employed FEM to predict sintering conditions, critical energy, and spatial distribution of electric fields, thermal responses, and densification in magnesium alloy compacts, seeking optimal thickness parameters for microwave sintering of hydroxyapatite materials [24]. Lu et al. conducted multiple simulations of the steel ring fabrication process to guide microwave sintering experiments, resulting in a 100 °C reduction in final sintering temperature compared to traditional methods, with improved Vickers hardness and fracture toughness [25]. Thuault et al. utilized FEM modeling to optimize experimental setups, successfully sintering large, complex-shaped kaolin in a microwave multimode cavity, reducing energy consumption by 82% compared to traditional sintering [26].

This study uses COMSOL (COMSOL Multiphysics 6.0) finite element simulation software to investigate the heating behavior of LiH in microwave fields. A physical model for LiH microwave heating is constructed, and the distribution of electromagnetic and thermal fields is simulated. By numerically simulating the microwave sintering process, this study reveals the temperature variation patterns of LiH in microwave fields and the impact of electromagnetic field distribution on the temperature field. To validate the accuracy of the simulation results, experimental comparisons were performed, analyzing the effects of conventional and microwave sintering processes on the internal structure of LiH. This research not only provides new insights into the physical behavior of LiH under microwave heating but also offers theoretical support and practical reference for optimizing the rapid densification sintering of LiH materials.

## 2. Experimental Methods

### 2.1. Materials and Procedures

In this study, LiH was prepared using a mature hydride method, and the resulting LiH powder was sieved into five samples of different particle sizes. LiH powder was subjected to cold isostatic pressing at 150 MPa for 3 min to produce the LiH pellets required for sintering. The conventional sintering temperature for LiH is 520 °C [27]. To encompass a broader range of possible sintering temperatures, the dielectric properties of the LiH samples were investigated using the resonant cavity perturbation method at a frequency of 2450 MHz over a temperature range from room temperature to 700 °C. Temperature is measured using a custom thermocouple (WRNK-191, Shanghai Instrument Group, Shanghai, China). The microwave heating behavior of LiH was studied using a custom-designed microwave tube furnace. The heating was conducted at a microwave frequency of 2450 MHz with a power of 2000 W under a controlled argon atmosphere throughout the process. Following heating, the microstructure of the samples was examined using a scanning electron microscope (JSM-5610, JEOL, Tokyo, Japan).

**Figure 1:**
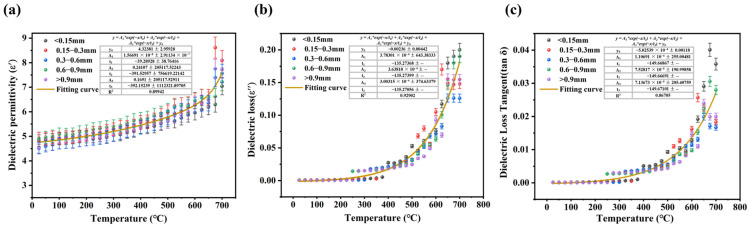
Variation of dielectric properties of LiH with temperature: (**a**) dielectric permittivity, (**b**) dielectric loss, and (**c**) dielectric loss tangent.

The effective response of a material to microwaves is a prerequisite for it to be heated by microwaves. Generally, materials that strongly respond to microwaves can be heated efficiently. The dielectric properties are key factors influencing the microwave absorption performance of materials. Among these, the dielectric permittivity, dielectric loss, and loss tangent are fundamental parameters used to evaluate dielectric properties [28,29]. Therefore, investigating the dielectric properties of LiH is essential for optimizing its microwave thermal processing. The dielectric properties of LiH powders with varying particle sizes were assessed using the resonant cavity perturbation method, with variations in dielectric properties depicted in Figure 1. Within the temperature range of room temperature to 700 °C, the dielectric permittivity, dielectric loss, and dielectric loss tangent of LiH increase with temperature. This increase is attributed to the enhanced crystallinity of the sample, which leads to a slight improvement in dielectric performance. The dielectric loss of LiH shows a gradual increase from room temperature to 400 °C, followed by a sharp rise beyond 400 °C. This behavior may be linked to a phase transition in LiH induced by elevated temperatures. The dielectric permittivity of LiH powders with different particle sizes exhibits complexity. Specifically, LiH powders in the particle size range of 0.6–0.9 mm demonstrate favorable wave-absorbing properties. The dielectric permittivity of LiH varies as a function of temperature, ranging from 4.561 to 8.553. The differences in dielectric properties among the powders of various sizes are not obvious; thus, we utilize the fitted results as working parameters for simulating the microwave heating of LiH.

### 2.2. Model Development

**Figure 2:**
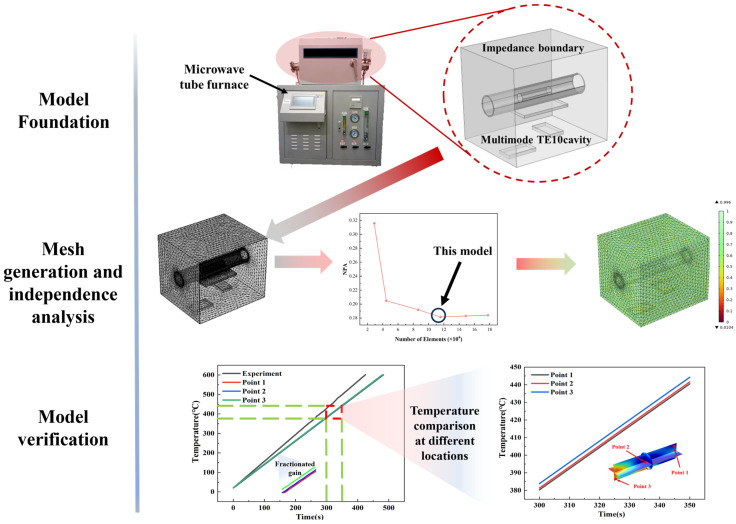
Model development, mesh generation, and validation for LiH microwave heating.

A detailed simulation analysis of LiH microwave heating behavior was conducted using the finite element analysis software COMSOL [30,31]. To ensure high consistency between the simulation conditions and experiments, as well as the accuracy of the results obtained, the constructed model was aligned in dimensions with the microwave tubular furnace used in validation experiments, as shown in Figure 2. Specifically, the dimensions of the microwave waveguide were set to 86 mm × 43 mm, utilizing a TEM10 transverse electric mode input. The heating chamber dimensions were 310 mm × 260 mm × 270 mm. In the simulation, the LiH sample dimensions were set to a diameter of 26 mm and a length of 100 mm. The sample was placed inside a quartz tube, which was positioned on a silicon carbide auxiliary heating plate within the heating chamber. The primary function of the quartz tube is to provide a stable, enclosed environment to prevent LiH from contacting external air, thereby avoiding oxidation and deliquescence reactions. Additionally, argon gas was introduced into the quartz tube to effectively prevent oxidation or other chemical reactions of the sample during high-temperature heating, ensuring the purity and controllability of the experiment. To accurately describe material properties during the simulation, key physical parameters such as the dielectric permittivity, thermal conductivity, and thermal expansion coefficient of LiH were dynamically measured under temperature variations during microwave heating. By inputting these temperature-dependent physical parameters into COMSOL, it was possible to accurately simulate the temperature distribution, heat flux variations, and other behaviors of LiH during microwave heating. This detailed parameterization allows for a comprehensive understanding of the thermal–physical characteristics of LiH under microwave heating conditions. Table 1 presents the structural parameters and selected operational parameters of the microwave equipment used in the LiH microwave heating model.

The interaction between physical fields during the microwave heating of LiH is exceedingly complex. Therefore, certain appropriate assumptions are necessary to simplify the problem. In this study, we established the following key assumptions for the LiH microwave heating model to investigate the distribution of the microwave field and temperature field during the heating process: 1. LiH is isotropic; 2. The initial temperature of LiH is uniform; 3. Gas flow within the cavity is negligible; 4. The solid–solid phase transition of LiH is ignored.

The model consists of two parts: microwave heating and solid heat transfer. The distribution of electromagnetic energy within the cavity is governed by the wave equation for the electric field [20,32,33], calculated as follows:

(1) $$\nabla \times \mu_{r}^{-1}\times \left(\nabla \times E\right)-k_{0}^{2}\left(\varepsilon -j\sigma /\omega_{0}\varepsilon_{0}\right)E=0$$

In the equation, ∇ stands for Hamiltonian operator, *E* denotes the electric field (V/m), *ε*_0_ represents the dielectric permittivity of vacuum, and *ε* denotes the relative dielectric permittivity of the medium. *μ_r_* is the relative permeability, *ω*_0_ is the angular frequency (rad/s), and *k*_0_ is the wave number in vacuum, while *σ* represents the electrical conductivity (S/m).

Temperature distribution is given by the heat transfer control equation [22], calculated as follows:

(2) $$\rho C_{p}\partial T/\partial t=k\nabla^{2}T+Q_{h}^{n0}$$

where *ρ* is the density of LiH (kg·m^−3^), *C_p_* is the specific heat capacity of LiH (J·kg^−1^·K^−1^), *T* represents temperature (K), t denotes time (s), and *k* denotes thermal conductivity (W·K^−1^·m^−1^), Q_h_^n0^ represents the converted heat source (w).

Mesh generation is a crucial step influencing computational accuracy and efficiency. In this study, a free tetrahedral mesh was used to discretize the entire geometric model to achieve reasonably accurate simulation results within a feasible computation time. The mesh size was meticulously set and optimized to balance accuracy with computational load. Firstly, mesh generation was performed for the microwave heating chamber and waveguide sections, as shown in Figure 2. To balance computational precision and efficiency, the maximum mesh element size for these regions was set to 15 mm. The appropriate number of grids is determined using normalized power absorption (NPA), defined as the power absorbed by the heating material divided by the effective input power, and the result is shown in Figure 2. A total of 376,773 tetrahedral elements were solved in the simulation, with the LiH sample occupying 115,529 of these elements. For the LiH sample, the maximum mesh size was 6.2 mm. This size configuration ensures that minute variations in temperature fields and heat flux distributions within the sample during microwave heating are captured in detail. Additionally, the refined mesh improves the accuracy of simulating physical parameter changes of LiH at different temperatures, enhancing the precision of the simulation results. After mesh generation, mesh quality was analyzed, as shown in Figure 2, demonstrating that the mesh generation supports the accuracy and convergence of the entire simulation process, ensuring accurate results within a relatively short computation time.

### 2.3. Model Validation

To ensure the accuracy and reliability of the simulation results for LiH’s microwave heating behavior, experimental measurements were compared with simulation results, as shown in Figure 2. In the experimental setup, thermocouples were used to precisely measure the temperature at the center of the material region. The thermocouples were placed at the central position of the LiH sample, allowing real-time monitoring of temperature variations during microwave heating. For the LiH model, three points were selected for comparison in the low, central, and high-temperature regions. The comparison indicated that the simulated temperatures were generally slightly higher than those measured experimentally. This discrepancy arises from multiple factors. On one hand, phase changes occurring in LiH during heating may not be fully captured by the simulation model, potentially missing complex thermodynamic variations during the phase transition. On the other hand, convective heat losses in the experimental environment, due to natural convection of surrounding air, result in partial heat dissipation to the environment, which was not fully considered in the simulation model, leading to higher simulated temperatures. Despite these differences, the overall trend of temperature curves in the simulation results shows a high degree of consistency with the experimental data. This trend consistency suggests that the simulation model accurately captures the thermal–physical behavior of the LiH sample during microwave heating.

## 3. Results and Discussion

### 3.1. Electric Field Analysis

**Figure 3:**
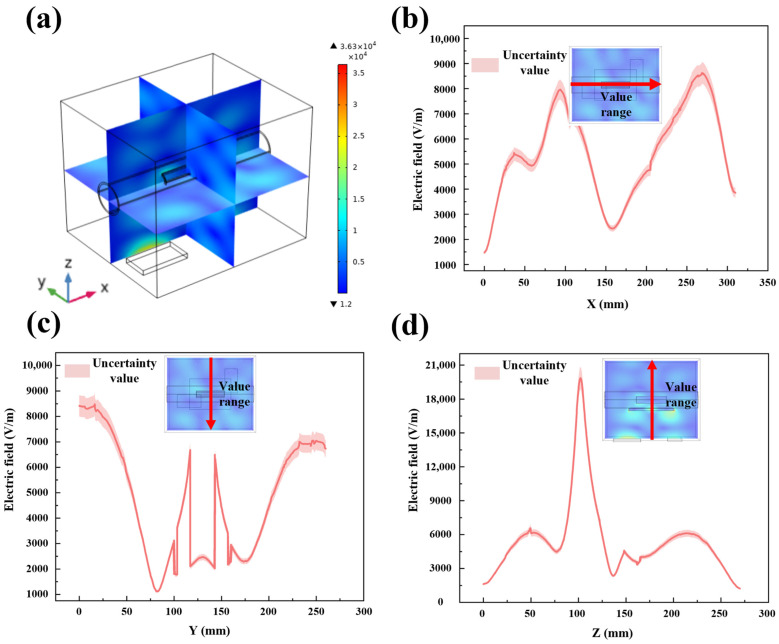
Simulation results of electric field distribution during microwave heating of LiH (**a**) distribution of electric field in cavity and sample; (**b**) distribution of electric field in X–Y plane along *X*-axis; (**c**) distribution of electric field in X–Y plane along *Y*-axis; (**d**) distribution of electric field in X–Z plane along *Z*-axis.

Electric field distribution is a critical factor in understanding the microwave heating process, as it directly affects the heating efficiency and temperature distribution of the material. Using finite element analysis software, we accurately simulated the propagation and reflection of microwaves within the heating chamber, waveguide, and LiH sample, revealing the distribution characteristics of the microwave electric field in different regions, as shown in Figure 3a. Once microwaves are activated, electromagnetic waves continuously reflect off the metal walls of the chamber and waveguide, creating a fluctuating electric field with alternating high and low energy regions. The simulation results provide a clear observation of the electric field distribution across various planes and regions. Figure 3b,c illustrate the two-dimensional electric field distribution along the X and Y axes through the chamber and LiH region, respectively. The electric field distribution ranges from 1472 V/m to 8621 V/m and 82 V/m to 8430 V/m, respectively. Figure 3d depicts the electric field strength distribution along the *Z*-axis through the center of the chamber. The waveguide is positioned at the bottom of the device, and the *Z*-axis plane represents a crucial cross-section for microwave propagation. Within the central *Z*-axis plane, the normal electric field strength varies from 1245 V/m to 19,814 V/m. It is observed that the electric field hotspots primarily occur in the middle region between the waveguide and the silicon carbide auxiliary heating plate. This is due to the formation of standing waves caused by the reflection of microwaves off the waveguide and chamber metal walls, resulting in higher electric field strengths in these regions [34,35]. In the LiH region, the electric field distribution is relatively uniform, indicating that the excellent microwave absorption properties of LiH results in a more even distribution of the electric field within the material. In the upper part of the chamber, a noticeable redistribution of the electric field occurs due to partial absorption of microwave energy by the LiH [36]. This phenomenon indicates that the energy distribution changes after passing through the LiH material, further validating the impact of microwave absorption properties of LiH on the electric field distribution.

**Figure 4:**
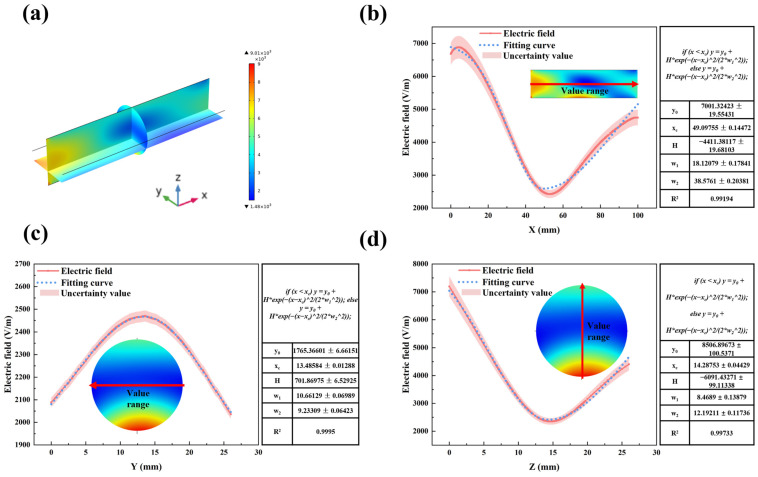
Electric field distribution in the LiH region during microwave heating of LiH and its fitting results (**a**) electric field distribution in the LiH region; (**b**) distribution of electric field in the Z–X plane along the *X*-axis; (**c**) distribution of electric field in the Y–Z plane along the *Y*-axis; (**d**) distribution of electric field in the X–Z plane along the *Z*-axis.

Figure 4 illustrates the two-dimensional electric field distribution along the *X*-axis, *Y*-axis, and *Z*-axis through the LiH region. The electric field distribution ranges from 2424 V/m to 6886 V/m along the *X*-axis, 2036 V/m to 2469 V/m along the *Y*-axis, and 2351 V/m to 7201 V/m along the *Z*-axis. It can be observed that there are no obvious electric field hotspots within the LiH region. This suggests that the dielectric properties of the microwave heating chamber and LiH contribute to a relatively uniform distribution of microwave energy within the LiH, thereby preventing localized overheating.

**Figure 5:**
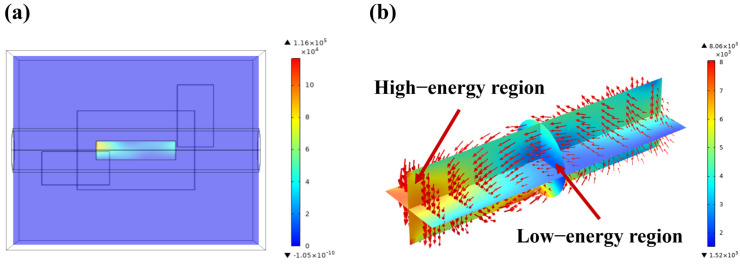
Simulation results of microwave heating of LiH: (**a**) distribution of resistive losses in the X–Y plane; and (**b**) magnitude and direction of electric field strength in the LiH region.

Figure 5 shows the distribution of resistive losses along the central X–Y plane. The figure shows that the resistive losses are negligible in the surrounding environment, with the primary resistive losses occurring within the LiH material. This is due to the fact that when current flows through LiH, a portion of electrical energy is transformed into thermal energy due to the resistive properties of LiH, as described by the coupling equation in Equation (2). The cavity is constructed from metallic materials, which act as microwave reflectors, reflecting rather than absorbing microwaves. Additionally, the surrounding environment consists of argon gas, a nonpolar molecule with a relatively low dielectric constant and a minimal loss factor, resulting in weak microwave absorption. Therefore, the distribution of resistive loss in the surrounding environment can be neglected. This indicates that, during microwave heating, the LiH material is the main area of energy absorption, with resistive losses concentrated within these regions [37]. Notably, the highest resistive losses, reaching 1.16 × 10^5^ W/m^3^, are observed in the regions of highest electric field strength within the LiH material. This results in a reverse heating gradient, where higher electric field strengths lead to greater absorption of microwave energy, producing more heat [38]. This heat then dissipates from the high-temperature regions of the material towards the periphery, creating a temperature gradient from the center outward. The redistribution of the electric field and the concentration of resistive losses during the heating process are critical factors influencing the overall heating effectiveness of the LiH material.

### 3.2. Temperature Field Analysis

**Figure 6:**
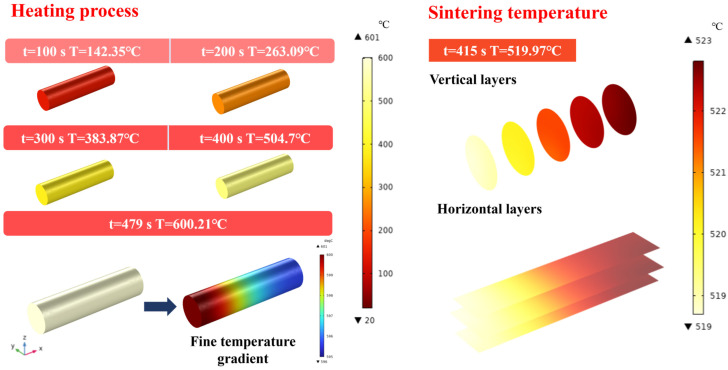
Temperature variation with time and temperature field distribution of LiH during microwave heating.

Figure 6 illustrates the temperature variation of LiH under microwave heating over time, with temperature measurements taken at 100 s intervals. As shown, LiH reaches the sintering temperature (520 °C) within 415 s, highlighting its rapid heating characteristics and the efficiency of microwave heating. Due to differences in the internal electromagnetic field of LiH, temperature distribution is not entirely uniform. Analysis of the temperature distribution within LiH at 479 s reveals a temperature difference of 5 °C between low- and high-temperature regions, with an overall relatively uniform temperature distribution.

**Figure 7:**
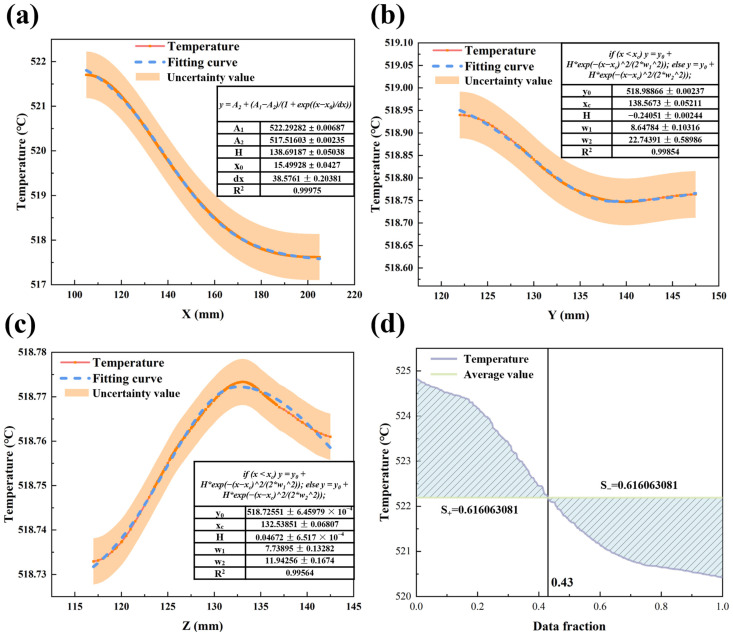
Simulation and its fitting results at 520 °C during microwave heating of LiH (**a**) distribution of Z-X plane electric field along the *X*-axis; (**b**) distribution of Y–Z plane electric field along the *Y*-axis; (**c**) distribution of X–Z plane electric field along the *Z*-axis; (**d**) distribution of the overall temperature field in the LiH region.

The sintering temperature affects the densification process of LiH. At 520 °C, the temperature distribution shows uniformity along the longitudinal direction of LiH, while there is a noticeable difference in the transverse distribution, with an overall temperature gradient of 4 °C, as shown in Figure 7. This phenomenon can be attributed to the concentration of microwave energy absorption in areas with higher electric field strength, leading to localized temperature increases. However, it is noteworthy that this temperature difference does not exacerbate with increasing overall temperature, as indicated in Figure 2. This suggests that LiH exhibits excellent uniform heating performance during the microwave heating process in the chamber.

To assess the overall temperature distribution in LiH, this study selected specific data points from the LiH material and plotted the corresponding figures in descending order of temperature, as shown in Figure 7d. The horizontal axis represents the percentage of selected data. The temperatures of each point were compared with the average temperature, and an area plot was generated. In an ideal temperature field distribution, the area of the plotted region should be close to zero. Analysis revealed that regions above the average temperature accounted for 43% of the LiH material, indicating that local high-temperature zones affect the temperature uniformity of LiH.

**Figure 8:**
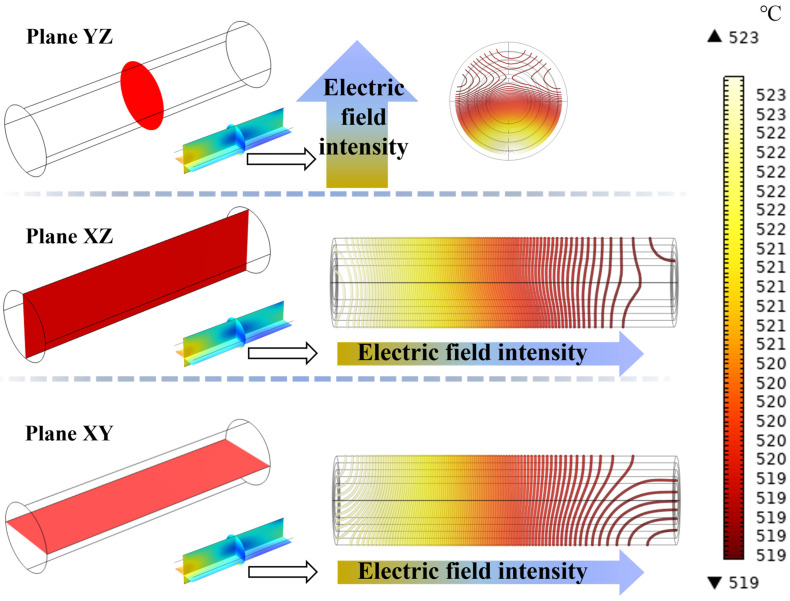
Contour map of isothermal lines for LiH at 520 °C.

Further analysis of the internal temperature distribution of LiH is shown in Figure 8, which presents isothermal contour plots of LiH at sintering temperature along three planes. It can be observed that isotherms are denser in regions with high electric field strength and become sparser in the direction of decreasing temperature gradient. Heat is conducted from areas of high electric field strength to areas of lower intensity, enhancing the uniformity of the internal temperature distribution of the material. Overall, the analysis indicates that microwave sintering not only facilitates rapid heating of LiH but also ensures uniform temperature distribution during the heating process, effectively achieving homogeneous densification of the material.

**Figure 9:**
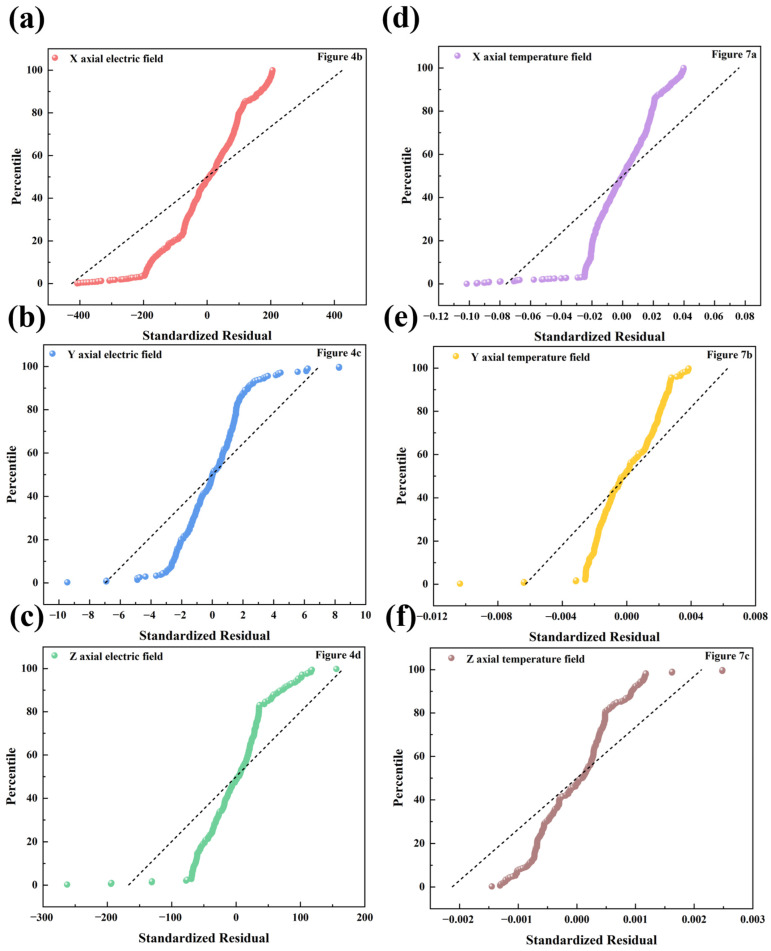
Normal probability plots of data fitted: (**a**) *X*-axis electric field; (**b**) *Y*-axis electric field; (**c**) *Z*-axis electric field; (**d**) *X*-axis temperature field; (**e**) *Y*-axis temperature field; and (**f**) *Z*-axis temperature field.

The fitting results for the LiH microwave heating model are summarized in Table 2, which includes R^2^, F-value, and reduced Chi-squared values. The R^2^ value indicates the degree of fit between the regression line and the observed data, with a maximum value of 1 signifying a perfect fit. The F-value assesses the significance of the overall fitting equation; a higher F-value indicates a more significant fit, while a lower F-value indicates a less significant fit. The reduced Chi-squared is a simplified chi-square test, where values closer to 0 are preferred, and a higher F-value indicates better fit. The fitting results appear to be appropriate, as indicated in the table. Figure 9 presents the normal probability plots for the model, which fit data from the *X*-axis electric field (a), *Y*-axis electric field (b), *Z*-axis electric field (c), *X*-axis temperature field (d), *Y*-axis temperature field (e), and *Z*-axis temperature field (f). It can be observed that the data closely cluster around the line, conforming to a normal distribution, which suggests that the model is suitable for simulating the microwave heating of LiH.

### 3.3. Experimental Analysis

**Figure 10:**
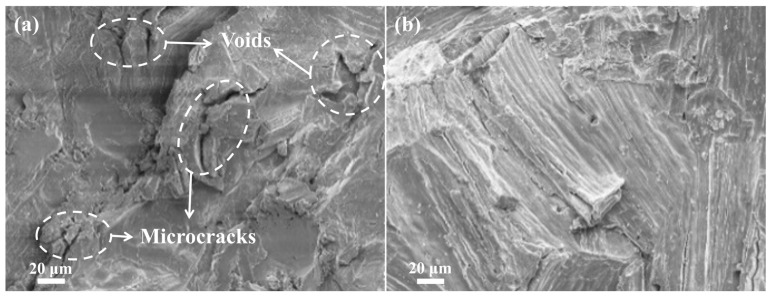
SEM images of LiH cross-sections after (**a**) conventional sintering and (**b**) microwave sintering.

Figure 10 compares the fracture surface morphology of LiH samples after conventional and microwave sintering, highlighting the effects of the two sintering methods on the material’s internal structure. The fracture surface of the conventionally sintered sample shows numerous voids and microcracks, with clear grain boundaries and a relatively loose crystal structure. These observations suggest that conventional sintering results in uneven heating, leading to obvious internal temperature gradients, insufficient material migration, and densification, ultimately resulting in higher porosity and lower material density. In contrast, the fracture surface of the microwave-sintered sample exhibits different characteristics. Microwave heating provides uniform heating, leading to a more consistent temperature distribution within the material and promoting thorough migration and reorganization of the material. The rapid and uniform heating achieved by microwave sintering effectively reduces internal temperature gradients, preventing localized overheating or uneven cooling. During microwave sintering, the interface migration within the LiH sample is notably enhanced. The grains gradually fuse under the influence of microwave energy, the grain boundaries become less distinct, voids are reduced, and tight metallurgical bonding is formed between grains. This demonstrates the advantages of microwave sintering in improving the uniformity and densification of the internal structure of LiH.

## 4. Conclusions

This study aimed to apply microwave heating for the sintering of LiH to mitigate the cracking issues commonly associated with traditional sintering methods. Through both simulation and experimental validation, the key findings and contributions of this research are summarized as follows:

1. The resonant cavity perturbation method was employed to characterize the dielectric properties of LiH across various temperatures. The results indicated that the dielectric permittivity increased from 4.561 at room temperature to 8.553 at 700 °C, accompanied by obvious rise in dielectric loss at elevated temperatures. This property facilitates efficient microwave energy absorption.
2. Finite element simulations using COMSOL revealed that LiH can reach its sintering temperature of 520 °C within just 415 s under microwave heating. This rapid heating capability is essential for enhancing processing efficiency.
3. The simulation results demonstrated that the electric field and temperature distributions within the LiH sample were uniform, effectively preventing localized overheating. The temperature differential during sintering remained below 5 °C, contributing to a stable thermal environment that reduces the likelihood of crack formation.
4. Experimental comparisons between conventional and microwave sintering showed that microwave sintering enhances grain fusion of LiH, leading to strong metallurgical bonding among grains. This highlights the distinct advantages of microwave sintering in improving material integrity and performance.

In conclusion, this research provides a theoretical framework and practical insights into the application of microwave heating for the sintering of LiH, emphasizing its potential to prevent cracking and enhance material properties. The findings contribute to the growing understanding of microwave sintering technology. It provides a theoretical basis and process optimization direction for the rapid densification of LiH materials.

## Figures and Tables

**Table 1:** Structural and operational parameters of the microwave equipment in the microwave heating model of LiH.

Parameter	Value
Length of microwave cavity	310 mm
Width of microwave cavity	260 mm
Height of microwave cavity	270 mm
Calibre of sample	26 mm
Length of sample	100 mm
Length of waveguide	86 mm
Width of waveguide	43 mm
Thickness of waveguide	20 mm
Outside radius of quartz tube	60 mm
Quartz tube thickness	5 mm
Microwave frequency	2.45 GHz
Microwave power	2000 W
Density of LiH	0.82 g/cm^3^
Initial temperature	20 °C

**Table 2:** Microwave heating simulation results for LiH.

Model		R^2^	F-Value	Reduced Chi-Sqr
Electric	X	0.992	34,164.99568	15,269.64978
	Y	0.999	130,278.76583	5.33818
	Z	0.997	25,136.5551	3037.96037
Temperature	X	0.999	1.30763 × 10^11^	4.96776 × 10^−6^
	Y	0.998	44,958.70908	4.31746 × 10^−6^
	Z	0.995	14,857.1033	4.96657 × 10^−7^

## Data Availability

The data that support the findings of this study are available from the corresponding author upon reasonable request. The data are not publicly available due to privacy or ethical restrictions.

## Nomenclature

ε′	Dielectric permittivity
ε″	Dielectric loss
Tanδ	Loss tangent
μ_r_	Relative magnetic permeability
E	Electric field (V/m)
k_0_	Vacuum wave number
ε	Relative dielectric permittivity
σ	Electrical conductivity (S/m)
ω_0_	Angular frequency (rad/s)
ε_0_	Vacuum permittivity
ρ	Density (kg·m^−3^)
C_p_	Specific heat capacity (J·kg^−1^·K^−1^)
T	Temperature (°C)
t	Time (s)
K	Thermal conductivity (W·K^−1^·m^−1^)
